# Antiproliferative and Apoptosis-inducing Effect of exo-Protoporphyrin IX based Sonodynamic Therapy on Human Oral Squamous Cell Carcinoma

**DOI:** 10.1038/srep40967

**Published:** 2017-01-19

**Authors:** Yanhong Lv, Jinhua Zheng, Qi Zhou, Limin Jia, Chunying Wang, Nian Liu, Hong Zhao, Hang Ji, Baoxin Li, Wenwu Cao

**Affiliations:** 1 Department of Anatomy, Harbin Medical University, Harbin, 150086, China; 2 Condensed Matter Science and Technology Institute, Harbin Institute of Technology, Harbin, 150080, China; 3 Materials Research Institute and Department of Mathematics, The Pennsylvania State University, University Park, Pennsylvania 16802, USA; 4 Department of Pharmacology, Harbin Medical University, Harbin, 150086, China; 5 Laboratory of Sono- and Photo-theranostic Technologies, Harbin Institute of Technology, Harbin, 150080, China

## Abstract

Sonodynamic therapy (SDT) is an innovative modality for cancer treatment. But the biological effect of SDT on oral squamous cell carcinoma has not been studied. Our previous study has shown that endo-Protoporphyrin IX based SDT (ALA-SDT) could induce apoptosis in human tongue squamous carcinoma SAS cells through mitochondrial pathway. Herein, we investigated the effect of exo- Protoporphyrin based SDT (PpIX-SDT) on SAS cells *in vitro* and *in vivo*. We demonstrated that PpIX-SDT increased the ratio of cells in the G_2_/M phase and induced 3–4 times more cell apoptosis compared to sonocation alone. PpIX-SDT caused cell membrane damage prior to mitochondria damage and upregulated the expression of Fas and Fas L, while the effect was suppressed if cells were pre-treated with p53 inhibitor. Additionally, we examined the SDT-induced cell apoptosis in two cell lines with different p53 status. The increases of p53 expression and apoptosis rate in wild-type p53 SAS cells were found in the SDT group, while p53-mutated HSC-3 cells did not show such increase. Our data suggest that PpIX-SDT suppress the proliferation of SAS cells via arresting cell cycle at G_2_/M phase and activating the extrinsic Fas-mediated membrane receptor pathway to induce apoptosis, which is regulated by p53.

Oral squamous cell carcinoma (OSCC) is a serious and growing problem in many parts of the world which accounts for more than 90% of all oral cancers^1^. After the traditional surgery treatment, survivors suffer significant long-term sequelae. Therefore, better methodologies are needed to improve the treatment of OSCC patients. The application of ultrasound in cancer treatment has attracted intensive interest^2^,^3^,^4^ due to its unique advantages of deep penetration depth compared to optical methods. It was also found that normal cells are more tolerant to ultrasonic sonication than malignant cells^5^. Although high intensity focused ultrasound therapy has shown the capability to directly kill cancer cells^6^,^7^, the intense implosions of cavitational bubbles and the induced high temperature could impose undesirable immune responses in the body. On the other hand, low intensity ultrasound (LIU) seems to cause rotations of certain intracellular organelles as well as to produce cell growth acceleration^8^,^9^.

Many *in vitro* and *in vivo* experiments had shown that sonodynamic therapy (SDT), i.e., LIU in combination with a sonosensitizer, could induce the death of tumor cells^10^,^11^,^12^,^13^. It was found that the death (especially apoptosis) of some cell lines and the reduction of tumor volume by ultrasound waves were much enhanced when some sonosentizers were used simultaneously^14^,^15^,^16^,^17^. Protoporphyrin IX (PpIX), as one of hematoporphyrin derivatives, is target-specific for cancer cells, while metabolize quickly in normal cells, which can serve as a sonosensitizer in the SDT treatment^15^,^18^,^19^. However, up to date the biomechnism of PpIX-based SDT is still not well understood.

Studies had demonstrated that PpIX-based SDT could trigger apoptotic response in murine tumor cell lines^19^,^20^. The simultaneous use of ultrasonic sonication and PpIX also damaged cytoskeletal F-actin in Ehrlich ascites carcinoma cells^21^. Some investigators hold the view that PpIX with ultrasound sonication mainly mediates mitochondria stress because the affinity of PpIX on the membrane of mitochondria^22^, while other experiments showed that the induced cellular damage by PpIX-based SDT appears to be mostly cell membrane related^19^,^23^ and is more effective than 5-Aminolevulinic acid (ALA)-based SDT^24^. These conflicting views indicate that there might be different mechanisms of SDT for different cell lines and different sonosensitizer, so that the biological mechanism of SDT needs further in-depth investigation. We have previously evaluated the cytotoxic effect of endo-PpIX (ALA) and LIU on human tongue squamous carcinoma SAS cell lines^25^,^26^,^27^, in which the enhancement of cell killing effect is partially through mitochondrion-mediated apoptosis signaling pathways. In this work, we investigated the effects of SDT on SAS cells *in vitro* and *in vivo* using exo-PpIX. The focus here is on cell cycle arrest, membrane receptor Fas-mediated cell apoptosis and the role of p53 in PpIX-based SDT induced anticancer effects.

## Methods

### Cell culture and tumor model

Two oral squamous cell carcinoma(OSCC)*-*derived cell lines (SAS and HSC-3, Human Science Research Resources Bank, Osaka, Japan) were grown in RPMI 1640 and Dulbecco’s Modified Eagle’s Medium (DMEM) with 10% fetal bovine serum (FBS) and cultured at 37 °C in a 5% CO_2_ humidified environment.

Male Balb/ca nude mice (SLAC; Shanghai Laboratory Animal Center, Shanghai, China) were housed in dedicated, pathogen-free barrier facilities. 0.2 mL suspensions of SAS cells at a density of 1 × 10^5^ cells/mL in serum-free medium were subcutaneously (s.c.) injected into the right flank of four-week-old mice. Treatment will begin when the tumor size of the mouse models reached about 0.3 cm to 0.4 cm in diameter after one week of inoculation. The animal is placed into a clear, plastic mouse restraint to make sure its transplantation tumor is able to be handled and exposed to ultrasound when they are treated. All animal experiments strictly followed the guidelines of and approved by the Laboratory Animal Committee of the Harbin Medical University.

### Sonication device and treatment protocol *in vitro* and *in vivo*

**Figure 1:**
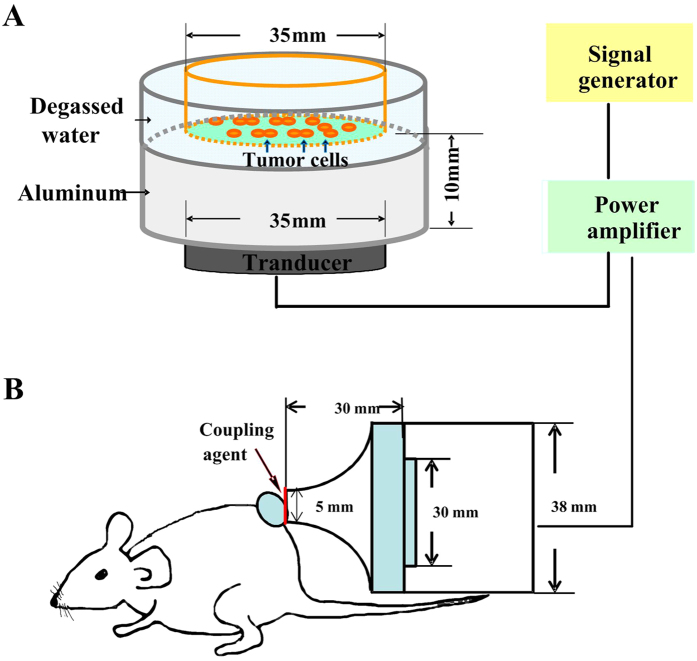
Schematic diagrams of ultrasound system for *in vitro* and *in vivo* experiments. (**A**) The ultrasonic transducer was fixed by aluminum stents facing upward. The culture dish was placed above the center of the transducer for the *in vitro* experiments. (**B**) The ultrasound signal was applied through a tapered aluminum head with its front surface directly in contact with the skin above the tumor site through coupling grease for the *in vivo* experiments.

The ultrasonic generator and power amplifier used in this study were designed and assembled by Harbin Institute of Technology (Harbin, China). For *in vitro* experiments, as shown in Fig. 1A, cells were paved in the vessel and put inside a water chamber and the cells were 10 mm away from the transducer surface. Sound pressure level distribution was calculated by finite element simulation using COMSOL as shown in Supplementary Figs S1 and S2. The ultrasound frequency was 1.0 MHz, provided in tone burst (TB) mode with a duty cycle of 10% and a repetition frequency of 100 Hz; ultrasonic intensity at this level was 0.12 W/cm^2^. Cell plate was floating and moving around slowly within the sound field when conducting sonication to make sure that all cells were exposed to the same amount of ultrasound energy. The SAS cells were divided into eight treatment groups: control (C), PpIX (Sigma Aldrich, St Louis, MO, USA) alone (P), sonication-1 min, 2 min, 3 min (U_1_, U_2_, U_3_), sonication-1 min, 2 min, 3 min plus PpIX (PU_1_, PU_2_, PU_3_). For the P and PU groups, the cells were incubated in the medium containing 10 μg/mL PpIX solution for 45 min in the dark.

Murine tumor treatment device is shown in Fig. 1B. The aluminum front of the transducer was placed directly on the tumor of the mice with coupling grease. Sound pressure level distribution is shown in Supplementary Figs S3 and S4. The ultrasound frequency was 1.0 MHz, provided in TB mode with a duty cycle of 20% and a repetition frequency of 100 Hz, the ultrasonic intensity level was 0.89 W/cm^2^. The tumor-bearing mice at a week after inoculation were randomized into four groups: the control group (C), PpIX solution alone (P), sonication alone (U), sonication plus PpIX (PU). Tumors in P and PU groups were injected locally with 10 μg/mL PpIX solution. Ultrasound was applied for 15 min in U and PU groups. All mice were treated daily and protected from light exposure until the end of the experiment.

### Assessment of cell viability *in vitro*

The instant viability and death of cells were determined by trypan blue exclusion test. 100 μL of cell suspension was taken and mixed with an equal volume of 0.4% Trypan blue solution (Sigma Aldrich, St Louis, MO, USA) and allowed to stand for 5 min at room temperature. The numbers of live and dead cells were counted by ECLIPSE TS100 optical microscope (Nikon, Tokyo, Japan) with a hemocytometer and quantified by the instant cell viability rate (%): (the number of intact cells/the number of total cells) × 100.

Long-term cell viability was measured using MTT cell proliferation assays. After various treatments, cells were re-incubated in 96-well plates for 2, 8, 14, and 20 h using a method described earlier^25^.

### Detection of cell cycle

After different treatments and incubation for 14 h, the cells were collected with a concentration of 1 × 10^6^ cells/mL and the suspension was detected with a flow cytometer, according to the instructions of the Cell Cycle Detection Kit (KeyGen Biotech, Nanjing, China). 1 mL cells suspension were fixed in 500 μL 70% ethanol, washed in PBS, and incubated at 37 °C for 30 min in the presence of 100 μL RNaseA (KeyGen Biotech), then mixed with 400 μL PI for 30 min (KeyGen Biotech). Each sample was analyzed by flow cytometry (FACSCount, NY, USA) at 488 nm excitation.

### Cell apoptosis analysis *in vitro*

Alexa Fluor^®^ 488 Annexin V/Dead Cell Apoptosis Kit (Invitrogen, OR, USA) was used for the apoptosis analysis according to the manufacturer’s instructions. SAS and HSC-3 cells were harvested after various treatments and a 14 h-incubation, washed three times with PBS and resuspended in 100 μL 1 × annexin-binding buffer. After adding 5 μL Alexa Fluor^®^ 488 Annexin V and 1 μL PI working solution to each cell suspension, the cells were incubated at room temperature for 15 min, then 400 μL 1 × annexin-binding buffer was added and the solution was analyzed immediately using a FC500 flow cytometer (Beckman Coulter Ltd., CA, USA).

To observe the apoptotic cells, the SAS cells were examined with a BX51 fluorescence microscope (Olympus, Tokyo, Japan) after Annexin V and PI double staining. The cells labeled with Annexin V-FITC were considered in early stage apoptosis, while those stained with both Annexin V-FITC and PI were considered in late stage apoptosis.

### Immunoblotting

Cells were lysed in RIPA buffer (Beyotime Biotechnology Inc., Nantong, China). Cell lysates (50 μg of protein) were separated by 10% SDS-PAGE, and electrophoretically transferred onto polyvinylidene fluoride membranes. After blocking in TBS-T containing 5% low-fat milk, the membranes were incubated with primary antibodies against the target proteins Fas (1:200), Fas L (1:200), caspase-8 (1:200) (Bioss Biological Technology, Ltd., Beijing, China); p53 (1:500, Boster Biological Technology, Ltd., Wuhan, China), with β-actin as a loading control. After washing twice with TBS-T, the membranes were incubated with fluorescence-conjugated goat anti-rabbit IgG secondary antibody (Invitrogen, CA, USA), and protein levels were detected using Odyssey infrared imaging system (LI-COR, Lincon, NE, USA). In experiments involving the p53 inhibitor (pifithrin α), the cells were pre-treated with 10 μM pifithrin α (Santa Cruz Biotechnology, Inc., CA, USA) prior to the ultrasound exposure.

### Immunofluorescence

The cells were fixed with 4% paraformaldehyde for 10 min and mixed 15 min with 0.5% Triton X-100 for permeabilizing after various treatments. Subsequently, cells were blocked with 1% BSA, followed by 2-hour incubation at 37 °C in the presence of the primary antibodies p53 and re-incubated for 1 h in the presence of a solution containing Cy3-conjugated secondary antibody. Cell nuclei were labeled with 5 μM PI. Finally, cells were mounted using FluorSave reagent and observed under the BX51 fluorescence microscope (Olympus). Protein expression was quantified with the integrated optical density (IOD) value using the software Image Pro Plus 6.0 (IPP 6.0) (Media Cybernetics, Inc., Bethesda, MD, USA).

### Evaluation of anti-tumor effect

The long and short diameters (a and b, in millimeters, respectively) of the tumors were measured daily with a slide caliper after the treatments. Tumor volumes were calculated according to the formula [(π/6) *a* × *b*^2^]. Body weight of the mice was measured every day. All mice were sacrificed and tumors were excised at the end of the treatment period.

### TUNEL

Apoptosis was assessed in xenograft tumors by using the terminal deoxyribonucleotide transferase-mediated nick-end labelling (TUNEL) method in combination with an *in situ* apoptotic detection kit (Boster Biological Technology, Ltd.) according to the manufacturer’s instructions, and stained with diaminobenzene (DAB) for 10 min. Slides were examined using a polarized light microscope (Nikon, Tokyo, Japan).

### Transmission electron microscopy

Xenografts were dissected and fixed with 2.5% glutaraldehyde for 2 h, post-fixed in 1% osmium tetroxide (OsO4) at 4 °C for 2 h, and embedded with Epon812 for 72 h at 60 °C. Ultra-thin sections were cut and stained with uranium acetate, followed by lead citrate, and then observed under a transmission electron microscope (TEM) (Hitachi, Tokyo, Japan).

### Immunohistochemical staining

Tumors were excised, fixed in 4% paraformaldehyde (PFA), dehydrated with a graded ethanol series, cleared in dimethylbenzene, and embedded in paraffin. Next, tissue blocks were cut into 4-μm sections by using a paraffin-slicing machine (Leica, Nussloch, Germany), and mounted on glass slides. Tissue sections were deparaffinized and rehydrated, heated in citrate buffer (0.01 M, pH 6.0), and treated with endogenous peroxidase at room temperature. After blocking in 10% goat serum, the sections were stained with rabbit polyclonal anti-Cyclin B_1_ (1:200) or mouse monoclonal anti-p53 (1:400) primary antibodies and incubated overnight at 4 °C. Subsequently, sections were incubated with secondary antibodies and stained with DAB reagent. Finally, all sections were observed under an electron microscope. Immunopositive expression in cells was quantified with IOD values by using IPP 6.0.

### Statistical analysis

All data were shown as mean values ± standard deviation. Differences between groups were assessed according to Student’s *t* test. Differences were considered statistically significant if *p* < 0.05.

## Results

### PpIX-based SDT significantly suppressed proliferation of SAS cells *in vivo* and *in vitro*

**Figure 2:**
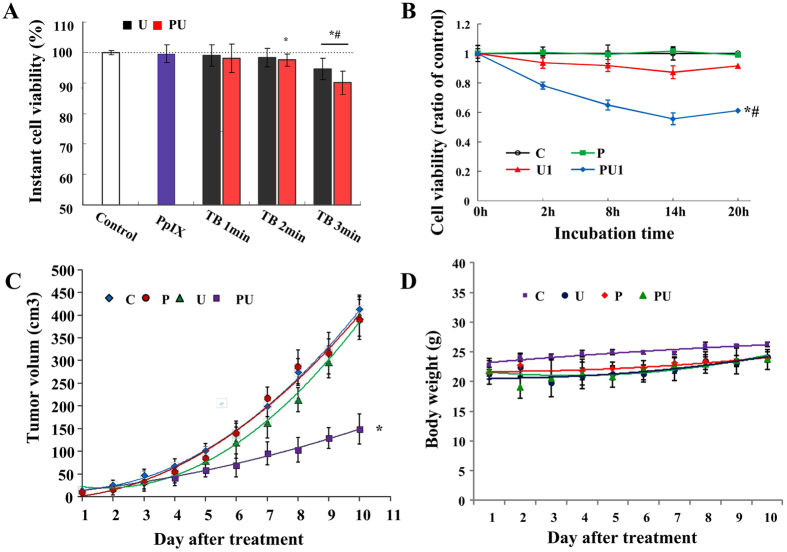
Growth inhibition of SAS cells *in vivo* and *in vitro.* (**A**) Instant cell viability evaluated by trypan-blue exclusion test after tone-burst ultrasound irradiation (TB) for 1, 2, or 3 min with or without PpIX. Cell viability was presented as a percentage of living cells relative to the control group and is represented as the mean ± SD of six experiments. *p < 0.05 *vs.* C, *^,#^*p* < 0.01 *vs.* C. (**B**) Long-term cell proliferation effect of different treatments, as tested by MTT assay at 2, 8, 14 and 20 h after treatments. Cell viability was presented as a percentage of absorbance at 570 nm relative to the control group. Error bars represent SD of the means (n = 5). *^,#^*p* < 0.01 *vs.* C, P or U_1_ group. (**C**) Effect of SDT on tumor growth *in vivo*. Average tumor volume observed after different treatments (n = 3). *p  <0.05 *vs.* C, P or U group. (**D**) Body weight in nude mice bearing SAS cell line xenogrfts after various treatments. Data are presented as the mean ± SD.

The instant viability rate of SAS cells after various treatments are shown in Fig. 2A. There is no obvious difference in the instant cell viability between the PpIX group and the control group (p = 0.598). Tone-burst ultrasound sonication 2 min alone or 1 min with PpIX did not cause any obvious changes in the instant cell survival rates compared to the control (p = 0.246 and p = 0.389). But cells exposed to ultrasound for 3 min alone or 2 min with PpIX displayed significantly difference in instant death rates compared to the control group (p < 0.05).

Since instant cell lysis is not what we want, tone-burst ultrasound for 1 min was selected for our MTT assays *in vitro* experiments to see if PpIX-based SDT has a long-term anti-proliferation effect on SAS cells. As shown in Fig. 2B, there is no obvious difference in cell viability between the C and P groups. The reduction of viability rate of SAS cells in the PU_1_ group is significant compared to that of the C group (p < 0.01). The lowest cell viability was observed in the PU_1_ group at 14 h incubation, for which the cell viability was 56.6%.

The anti-tumor efficacy of PpIX-based SDT was also evaluated *in vivo*. The tumor growth in different treatment groups was evaluated by measuring the tumor volume. As shown in Fig. 2C, PpIX or ultrasound alone at the selected low dosages did not affect the tumor growth, but ultrasound combined with PpIX inhibited the tumor growth by more than 3 times. Furthermore, no death or adverse effects, such as skin ulceration, were observed in any of the animal groups. There was no difference in body weight among different treatment groups (Fig. 2D).

### PpIX-based SDT induced G_2_/M arrest

**Figure 3:**
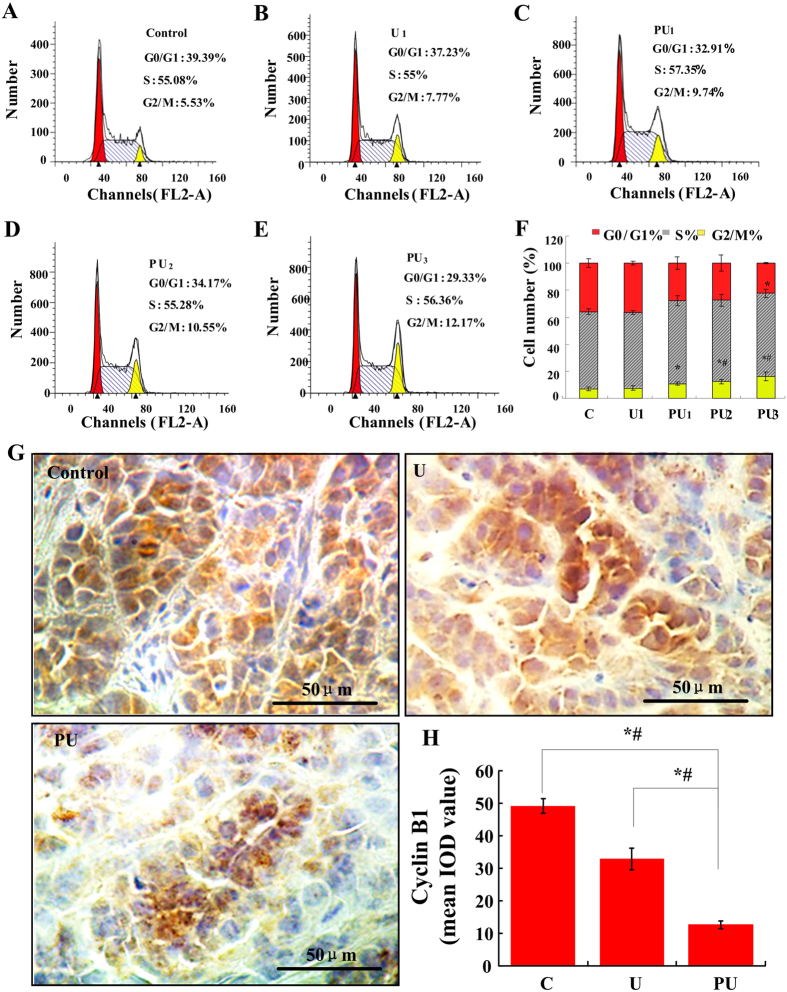
Cell cycle analysis of SAS cells after different treatments. (**A–E**) Representative flow cytometry images exhibiting changes in the progression of cell cycle of different groups. (**F**) Histogram represents the percentage of cells arrested in different phases of cell cycle. Error bars represent SD of the means (n = 3). *p < 0.05 vs control or U_1_ group, *^,#^*p* < 0.01 vs control or U_1_ group. (**G**) Effect of PpIX-based SDT on G_2_/M phase specific protein Cyclin B_1_ in SAS cells *in vivo.* Immunohistochemical analysis *in vivo* shows cytoplasmic staining for Cyclin B_1_ in different treatment group. Bar: 50 μm. (**H**) The expression level of Cyclin B_1_ was evaluated for *in vivo* experiments using the mean IOD value. Data are presented as the mean ± SD values. *^,#^*p* < 0.01 vs. C and U group.

Blocking cell-cycle progression is one of the ways to inhibit tumor development. SAS cell percentages in the G_0_/G_1_, S and G_2_/M phases after various treatments are showed in Fig. 3A,B. Cells treated with ultrasound alone had no obvious cell cycle changes compared to that of the control group. However, the percentage of SAS cells in PU_1_, PU_2_ and PU_3_ groups decreased in the G_0_/G_1_ phase, increased in the S phase and increased more obviously in the G_2_/M phase compared to that of the control group (p < 0.05, p < 0.01 and p < 0.01).

Furthermore, we detected the expression of the G_2_/M phase arrest related protein Cyclin B_1_ by immunohistochemical staining *in vivo*. The above proteins were expressed in the cytoplasm of each group (Fig. 3C). Protein expression levels were assessed by the mean IOD value of brown positive particles. The expression of Cyclin B_1_ in the PU group was about 1/4 of that of C group (p < 0.01, Fig. 3D).

### PpIX-based SDT induced SAS cells apoptosis

**Figure 4:**
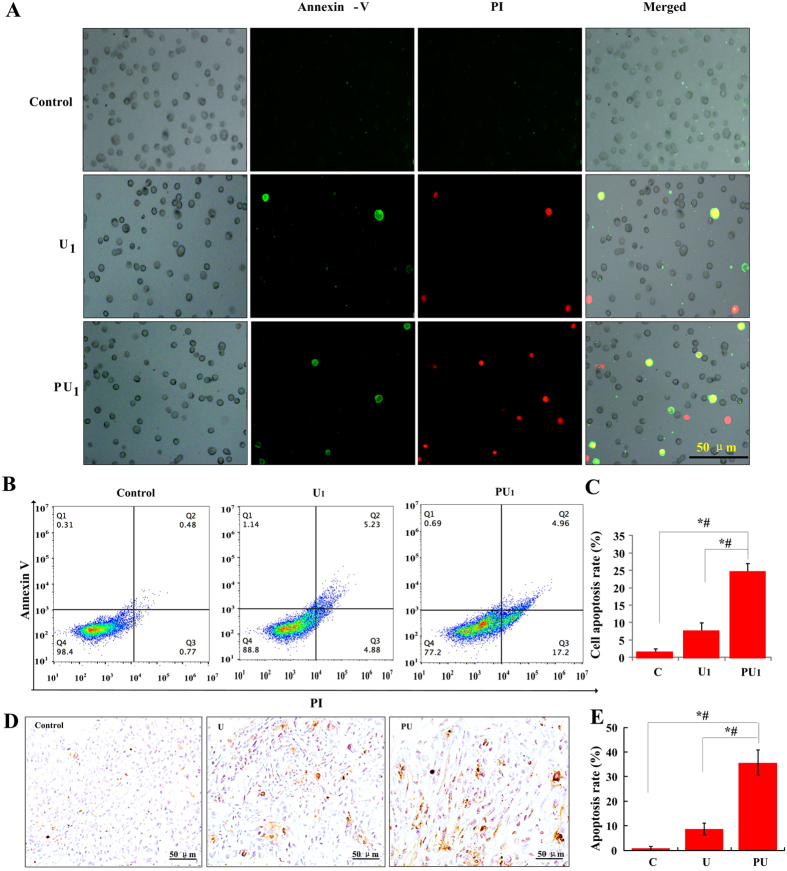
Apoptosis induction with PpIX-based SDT or LIU *in vitro* and *in vivo.* (**A**) Fluorescence microscopy images of apoptotic SAS cells subjected to Annexin V-PI double staining *in vitro*. Annexin V-FITC in conjunction with PI staining could distinguish early apoptotic (Annexin V-FITC-positive cells yielded green fluorescence) from late apoptotic (Annexin V-positive and PI-positive cells yielded green and red fluorescence, respectively) cells. Bar: 50 μm. (**B**) Representative imagines of FCM assay showed the effect of apoptosis induced by PpIX-based SDT on SAS cells. (**C**) The rate of apoptosis of SAS cells determined by FACS assay *in vitro*. *^,#^*p* < 0.01 vs. C and U_1_ group. (**D**) Representative images of TUNEL staining of apoptotic cells (brown, pointed by black arrows) in tumor tissue. Bar: 50 μm. (**E**) The cell apoptosis rate *in vivo* was calculated according to the formula: number of positive-stained cells/number of total cells. *^,#^*p* < 0.01 vs. C and U group.

The other way of tumor inhibition is to induce cell death. SDT induced apoptosis cells were observed through fluorescent microscopy (Fig. 4A). The cell membrane and nucleus were stained with Annexin-V and PI into green and red, respectively. Both early and late stage apoptosis cells increased in the PU_1_ group. Figure 4B,C showed the apoptosis rates of SAS cells after different treatments, the apoptosis rate of PU_1_ groups is about 3 and 14 times of that of the U_1_ and C group, respectively.

Subsequently, we detected the cell apoptosis induced by PpIX-based SDT *in vivo* using TUNEL assay. As shown in Fig. 4D, apoptosis rate (brown stained cells) increased in the PU group, which is 35.61% but is only 8.72% in the U group (Fig. 4E).

### SDT induce extrinsic membrane death receptor-mediated apoptosis pathway

**Figure 5:**
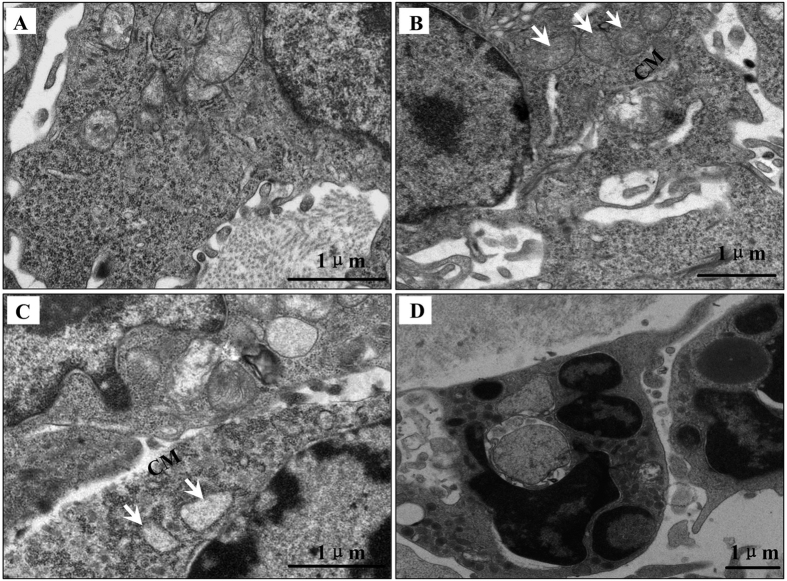
Transmission electron microscopy images of ultrastructural changes in SAS cell xenograft tissue after PpIX-LIU treatment. (**A**) Tumor cells in untreated tissue. (**B**) Tumor cells show intact mitochondria (white arrows) and destroyed cell membrane (CM). (**C**) Mitochondria (white arrows) inside the cell were vacuolated, other organelles were destroyed and cell membrane was damaged (CM). (**D**) Apoptotic bodies. Bar: 1 μm.

TEM showed that untreated cells displayed ultrastructure characterized by intact cell membrane, rich and polar distributed microvilli, and normal structure of organelles (Fig. 5A). In the PU group, some cells exhibited damaged cell membrane with reduced or even disappeared microvilli (Fig. 5B), but kept mitochondria intact; some cells exhibited both damaged membrane and damaged organelles (Fig. 5C); and other cells formed apoptotic bodies, in which the surface of nuclear membranes became lumpy with a condensed heterochromatin (Fig. 5D).

**Figure 6:**
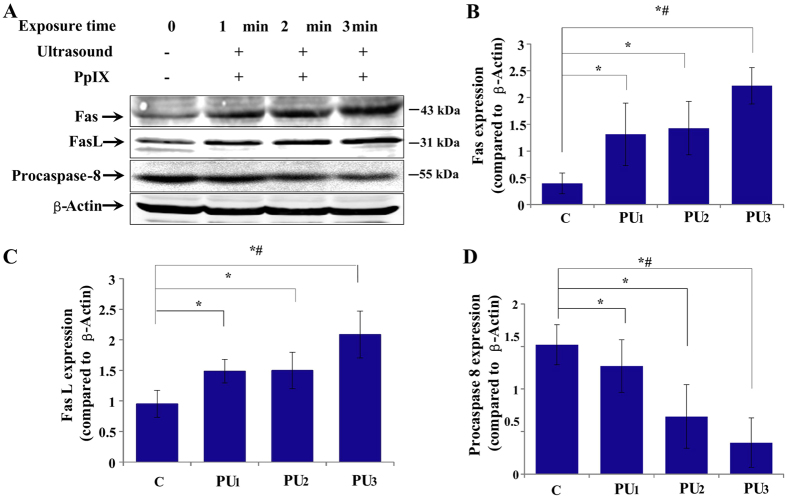
Changes in the expression of Fas, Fas L and procaspase-8 after various treatments were analyzed by immunoblotting *in vitro*. (**A**) Representative images of immunoblotting in various groups. (**B–D**) The amount of each protein was normalized to β-actin. The data are expressed as the mean ± SD (n = 3), *p < 0.05 and *^,#^*p* < 0.01 *vs* C group. Increased expression of Fas and Fas L and decreased expression of procaspase-8 were observed in an ultrasound-exposure-time dependent manner in the PU treatment groups.

Ultrastructural evidence suggests that there is an obvious damaged cell membrane and the change happened prior to the damage of mitochondria. Previous study has shown that exogenous PpIX was mainly distributed in cell membranes^24^, the subcellular sites localized sonosensitizers, such as plasma membrane and mitochondria, are often considered main targets for SDT treatment^28^,^29^. Therefore, we hypothesized that cell membrane is the main target of PpIX-based SDT. Following this assumption, we examined the expression of membrane-associated apoptotic receptor Fas, its ligand Fas L and procaspase-8 by western blot analysis *in vitro*. The expressions of Fas and Fas L were increased 5 and 2 times, respectively, and the procaspase-8 was decreased by 80% in the PU_3_ group compared to the C group. The results also revealed an ultrasound-treatment-time dependent manner in the level of these proteins (Fig. 6).

### Involvement of p53 in SDT induced death receptor-mediated apoptosis

**Figure 7:**
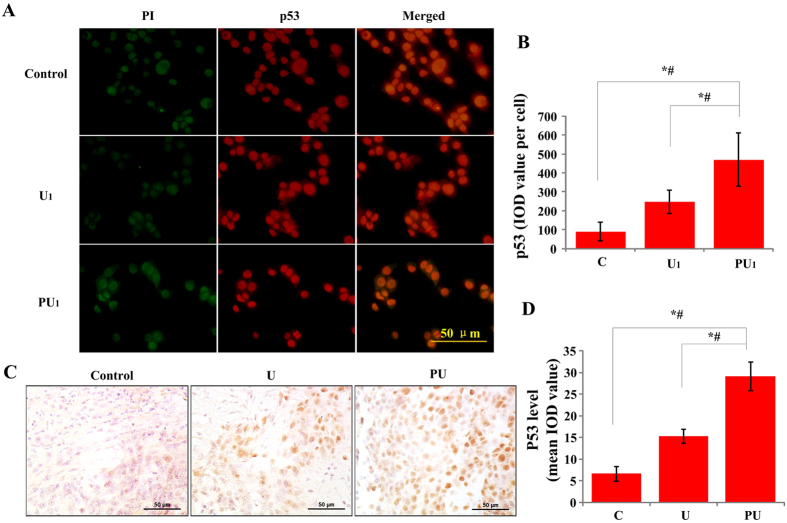
Effect of PpIX-based SDT on p53 protein in SAS cells *in vivo* and *in vitro*. (**A**) Immunofluorescence staining was used to detect the expression of p53 in various groups *in vitro*, p53 stained (shown in green), PI stained (shown in red) and p53-PI images merged. Bar: 50 μm. (**B**) Integrated optical intensity values of p53 immunostaining after different treatments. Error bars represent SD of the means (n = 5). *^,#^*p* < 0.01 vs C or U_1_ group. (**C**) Immunohistochemical staining for p53 protein expression *in vivo*. Bar: 50 μm. (**D**) The expression level of p53 protein of *in vivo* experiment was evaluated using the mean IOD value. Data are presented as the mean ± SD values. *^,#^*p* < 0.01 *vs.* C or U group.

It is well known that p53 gene plays a vital role in cell cycle arrest, apoptosis and restoration^30^. As a transcription activating factor, p53 could target death receptor apoptotic pathway^31^. We found that PpIX-based SDT could induce G_2_/M phase arrest and induce apoptosis of SAS cells. The question is: if the p53 is involved in the antitumor effect of PpIX-based SDT. Immunofluorescence staining *in vitro* of p53 distribution is shown in Fig. 7A, p53 mainly expressed in the nuclei of the control group, while in the PU_1_ group, the proteins are expressed in the nuclei and cytoplasm. The expression level of p53 in the PU_1_ group increased compared to that of the C or U_1_ group (p < 0.05) (Fig. 7B). Similar results were achieved by immunohistochemical staining *in vivo*, which also revealed that the expression of p53 increased significantly in the PU group than that in the C or U groups (Fig. 7C,D).

**Figure 8:**
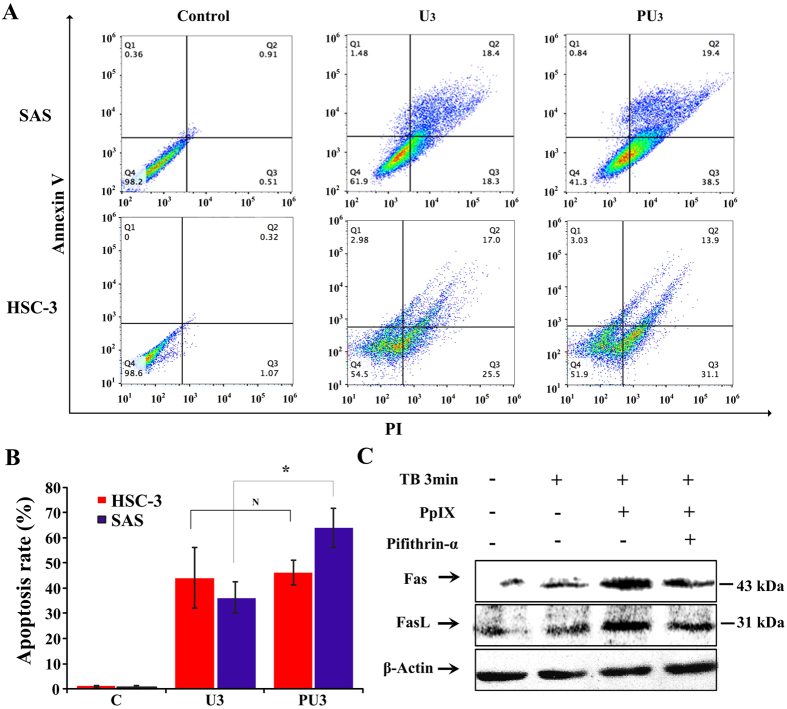
The tumor suppressor p53 involved in SDT induced cell membrane death receptor Fas-Fas L-mediated extrinsic apoptosis pathway. (**A**) Representative imagines of FCM assay showed the effect of apoptosis induced by PpIX-based SDT on wild-type p53 SAS cells and p53-mutated HSC-3 cells *in vitro*. (**B**) The rate of apoptosis of SAS cells determined by FCM assay *in vitro*. N represents no statistically significant difference between groups, *p < 0.05 between groups. (**C**) Effect of p53 inhibitor Pifithrin-α on protein activity *in vitro*. PpIX-based SDT induced the activation of Fas and Fas L, while Pifithrin-α substantially suppressed such effect.

To further confirm the relationship between p53 and pro-apoptosis effect of PpIX-based SDT, we detected apoptosis rate of two cell lines: wild-type p53 SAS and p53-mutated HSC-3 cell line. FCM analysis revealed that there was no obvious difference in the total apoptosis rate of HSC-3 cells between the U_3_ and PU_3_ group, but a significant difference (p < 0.05) was found between the two groups in SAS cells (36.2% ± 6.23% vs. 63.9% ± 7.8%) as shown in Fig. 8A and B. Wild-type p53 SAS cells showed a marked increase in the total cell apoptosis rate in the PU_3_ group.

Subsequently, the effects of p53 inhibitor (Pifithrin-α) on the expressions of Fas and FasL in the PpIX-based SDT treated group were further examined by western blot. The expressions of Fas and Fas L increased in the PU group compared to that of the C group and the effect is substantially reduced when the cells were pre-treated with Pifithrin-α (Fig. 8C).

## Discussions

Tumor treatment using PpIX-based SDT has been extensively investigated in term of anti-proliferation effects. PpIX, a hematorphyrin derivative, was found preferentially accumulated in rapid proliferating cancer cells, and it is sonosensitive^18^,^32^,^33^,^34^. In previous studies, we showed that ALA can be a good sonosensitizer for SDT as it exhibited significant selectivity on tumor tissues and a synergistic inhibition effect with low intensity ultrasound on SAS cells^25^,^26^,^27^,^35^,^36^. In this work, we demonstrated, both *in vivo* and *in vitro*, that the combined use of PpIX and LIU can significantly inhibit the proliferation of SAS cells, which acted through the cell cycle arrest and p53-dependent extrinsic apoptosis pathway.

In general, different ultrasonic parameters, including intensity, frequency, wave form, duty cycle etc., could lead to different cell death patterns via different mechanisms. Direct killing and inducing apoptosis are two anti-tumor ways of ultrasound. Low intensity ultrasound mainly induces cell apoptosis when combined with a sonosensitizer, while high intensity ultrasound mainly induces necrosis. High intensity ultrasound in liquid leads to acoustic cavitation and the collapsing of cavitational bubbles, which can destroy cell membranes, leading to direct killing of cells^3^. However, such direct cell death always produces undesirable immune reactions *in vivo* in cancer therapy, which is undesirable. In our study, tone-burst ultrasound (0.12 W/cm^2^, 10% duty cycles) duration of 1 min combined with PpIX did not increase the instant cell death rate but showed a prolonged antitumor activity *in vitro*. Tone-burst ultrasound (0.89 W/cm^2^, 20% duty cycles) combined with PpIX *in vivo* could effectively inhibit the growth of SAS cell xenografts in nude mice.

There were limited studies in the literature on SDT inducing cell cycle changes. Li *et al*. found that 0.5 W/cm^2^ ultrasound plus HMME increased the percentage of C6 cells in the S phase^37^. High energy shock waves combined with ALA were able to induce HT-29 cells arrest in the G_0_/G_1_ phase^38^. We found that cell cycles were arrested at the G_2_/M phase in the group treated by PpIX-based SDT and the effect is ultrasound treatment time dependent *in vitro*. Also, the expression level of G_2_/M related protein Cyclin B_1_ decreased after the SDT treatment *in vivo*. Our results clearly show that anti-proliferative effect of PpIX-based SDT on SAS cells is associated with the G_2_/M arrest.

As observed by some experiments, the tumor inhibitory activity of SDT on tumor cells was mainly to induce cell apoptosis^14^,^35^,^39^,^40^. The pro-apoptotic effect and related bio-mechanisms vary with the type of sonosensitizers used in cancer treatment. Some studies showed that PpIX or ALA combined with ultrasound of certain parameters could active the mitochondria apoptosis pathway, Fas-dependent apoptosis or PARP involved apoptosis in different cells^19^,^36^,^41^,^42^. Our previous study showed that ALA-based SDT induce SAS cell damage in part through mitochondrion-mediated apoptosis pathway, apoptotic rate of SAS cells increased in an ultrasound sonication time-depended manner^25^. In this study, tone-burst ultrasound (0.12 W/cm^2^, 10% duty cycles) combined with PpIX induced less lysis but more apoptosis cells. TEM results *in vivo* not only provided the morphological evidence of apoptosis, but also indicated the fact that cell membranes changed prior to the mitochondrial vacuolization. The cell apoptosis signals probably originated at the plasma membrane. Furthermore, we found that the expression levels of extrinsic membrane-associated death receptors Fas and its ligand Fas L increased in PpIX-based SDT, while the procaspase-8 decreased, both also in an ultrasound exposure time-dependent manner. It was demonstrated that the expressions of Fas and Fas L have a positive correlation with cell apoptosis, exo-PpIX based SDT induced SAS cell apoptosis probably through extrinsic apoptosis pathway, i.e., the death receptor Fas pathway.

The tumor suppressor p53 relates to both cell cycle arrest and apoptosis, which was studied only in hematoporphyrin, HMME and ALA-based SDT^36^,^43^,^44^. Our results showed that the expression of p53 was upregulated after PpIX-SDT in SAS cell line. There is no significant difference on apoptosis rates between the SDT group and ultrasound alone group in mutant-p53 HSC-3 cell line but very obvious difference in wild-type-p53 SAS cell line. These results indicated that p53 plays a vital role in PpIX-based SDT, which regulates cell apoptosis and induces cell apoptosis through death signal receptor proteins, such as the Fas proteins^31^. Furthermore, we found that the expressions of Fas and Fas L decreased in the SAS cells when they were pretreated with p53 inhibitor compared to the non-inhibitor SDT group. These results showed that PpIX-based SDT could activate the Fas-Fas L-mediated apoptosis pathway, which was, at least partially, regulated through p53.

In conclusion, PpIX-based SDT can effectively suppress the growth and proliferation of tongue carcinoma SAS cells through cell cycle arrest and inducing cell apoptosis. The apoptotic mechanism of exo-PpIX-based SDT differs from that of endo-PpIX (ALA) -based SDT in SAS cells. The former begins with mitochondrial damage, while the latter is through blocking the G_2_/M phase of cell cycle, active the extrinsic Fas-mediated cell membrane receptor pathway to induce apoptosis, which was partially regulated by p53.

## Additional Information

**How to cite this article**: Lv, Y. *et al*. Antiproliferative and Apoptosis-inducing Effect of exo-Protoporphyrin IX based Sonodynamic Therapy on Human Oral Squamous Cell Carcinoma. *Sci. Rep.* **7**, 40967; doi: 10.1038/srep40967 (2017).

**Publisher's note:** Springer Nature remains neutral with regard to jurisdictional claims in published maps and institutional affiliations.

## Supplementary Material

Supplementary Dataset 1
