# Supplementary material for: Antiproliferative and Apoptosis-inducing Effect of exo-Protoporphyrin IX based Sonodynamic Therapy on Human Oral Squamous Cell Carcinoma
Source: Sci Rep. 2017 Jan 19;7:40967. doi: 10.1038/srep40967 (PMC5244424; doi:10.1038/srep40967)
Supplement: Supplementary Dataset 1 [file srep40967-s1.doc]

**Antiproliferative and Apoptosis-inducing Effect of exo-Protoporphyrin IX based Sonodynamic Therapy on Human Oral Squamous Cell Carcinoma**

Yanhong Lv^1,#^, Jinhua Zheng^1,#^, Qi Zhou^2^, Limin Jia^1^, Chunying Wang^2, 3^, Nian Liu^1^, Hong Zhao^1^, Hang Ji^1^，Baoxin Li^4, *^ & Wenwu Cao^2,3,5,*^

1. Department of Anatomy, Harbin Medical University, Harbin, 150086, China

2. Condensed Matter Science and Technology Institute, Harbin Institute of Technology, Harbin, 150080, China.

3. Materials Research Institute and Department of Mathematics, The Pennsylvania State University, University Park 16802, USA

4. Department of Pharmacology, Harbin Medical University, Harbin, 150086, China.

5. Laboratory of Sono- and Photo-theranostic Technologies, Harbin Institute of Technology, Harbin 150080, China

^*^Corresponding to: Prof. Wenwu Cao, Prof. Baoxin Li

217 Materials Research Lab, The Pennsylvania State University, University Park, PA, 16802

Tel: +1-814-8654101

Fax: +1-814-8652326

E-mail: dzk@psu.edu

**Supplementary Figures**


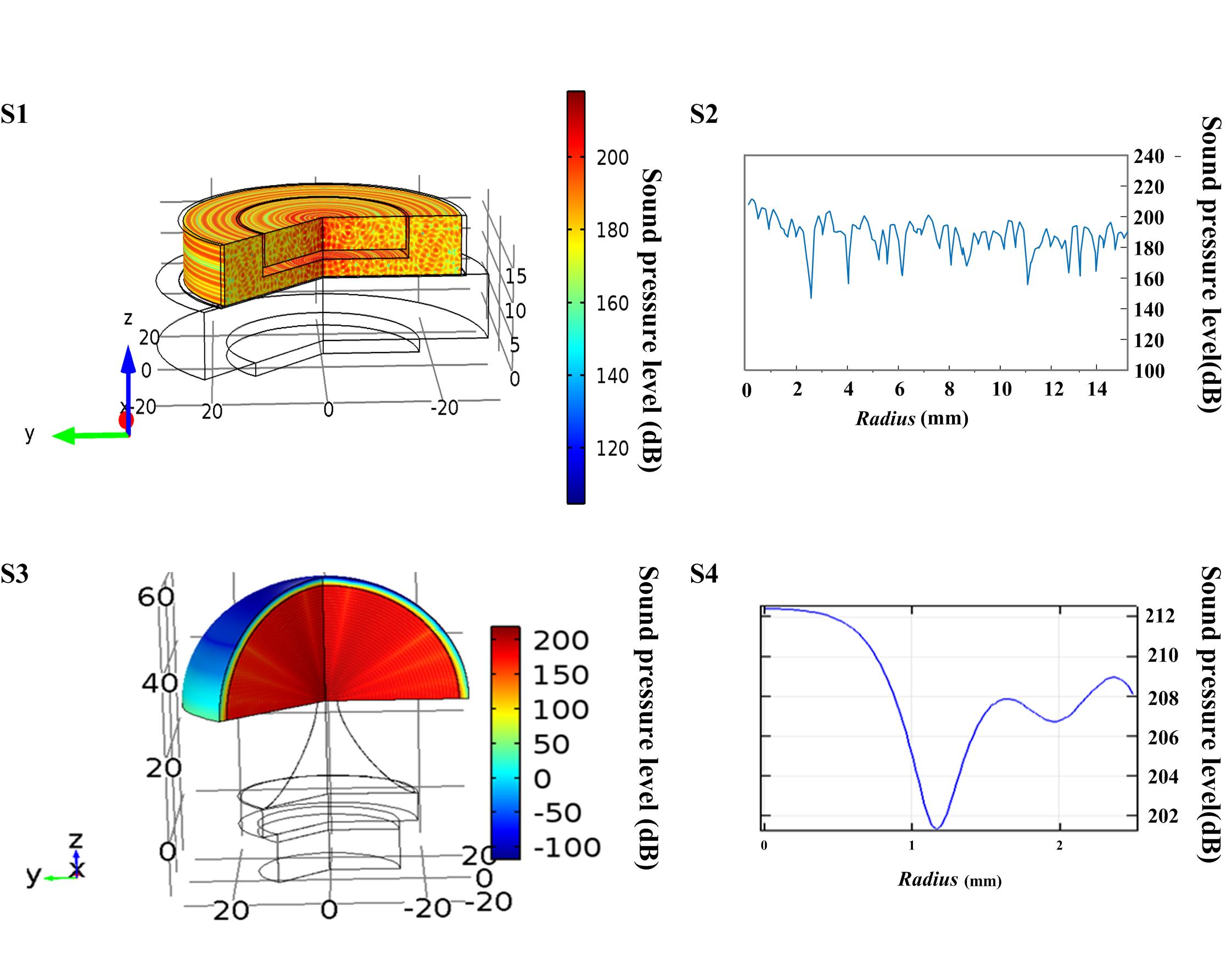


**Figure Legends**

**Supplementary Figures:** Schematic diagrams of ultrasound pressure level distribution for *in vitro* and *in vivo* experiments.

(S1) The sound pressure level distribution for *in vitro* experiments. (S2) The sound pressure distribution at the horizontal plane of cells. (S3) The sound pressure level distribution for *in vivo* experiments. (S4) The sound pressure distribution at the tumor surface.
